# *Drosophila ppk19* encodes a proton-gated and mechanosensitive ion channel

**DOI:** 10.1038/s41598-022-23236-3

**Published:** 2022-11-01

**Authors:** Wijeong Jang, Ji Yeon Lim, Seyoung Kang, Minseok Kim, Sun Wook Hwang, Changsoo Kim

**Affiliations:** 1 School of Biological Sciences and Technology, Chonnam National University, Gwangju, 61186 Korea; 2 Department of Biomedical Sciences and Department of Physiology, Korea University College of Medicine, Seoul, 02841 Korea

**Keywords:** Genetics, Neuroscience

## Abstract

In *Drosophila* larvae, nociceptive mdIV sensory neurons detect diverse noxious stimuli and prompt a nociceptive rolling response. Intriguingly, the same neurons also regulate stereotyped larval movement. The channels responsible for transducing these stimuli into electric signals are not yet fully identified. Here we undertook genetic and electrophysiological analysis of Ppk19, a member of the Deg/ENaC family of cationic channels. *ppk19* mutants exhibited an impaired nociceptive rolling response upon mechanical force and acid, but no impairment in response to noxious temperature and gentle touch. Mutants also exhibited defective larval movement. RNAi against *ppk19* in mdIV neurons likewise produced larvae with defects in mechanical and acid nociception and larval movement, but no impairment in detection of heat and gentle touch. Cultured cells transfected with *ppk19* produced currents in acid and hypotonic solution, suggesting that *ppk19* encodes an ion channel that responds to acid and cell swelling. Taken together, these findings suggest that Ppk19 acts in mdIV neurons as a proton- and mechano-gated ion channel to mediate acid- and mechano-responsive nociception and larval movement.

## Introduction

In animals, peripheral sensory nociceptive neurons (nociceptors) express ion channels that detect noxious stimuli and transduce nociceptive signals into neuronal firing, inducing an aversive response. The nocifensive behavior of *Drosophila* larvae is a stereotyped rolling that occurs upon exposure to noxious heat, harsh mechanical force, irritating chemicals, and, as was recently reported, acid^1–3^. This rolling response is mediated by larval sensory nociceptive neurons, the multidendritic class IV (mdIV) neurons, which—much like mammalian nociceptors—tile under the skin across the whole body and feature naked dendrites^1,4^. Intriguingly, mdIV neurons also regulate larval movement, which is a stereotyped crawl and turn movement^5^. The ion channels in mdIV neurons that are responsible for detecting noxious acid and mechanical stimuli have yet to be fully identified.

Pickpocket1 (Ppk1), a member of the Deg/ENaC family of cationic ion channels, is known to be specifically expressed in sensory nociceptors (mdIV), wherein it plays a role in larval movement^5,6^. Intriguingly, it is also involved in mechanical nociception^7^. Proteins of the Ppk family (and the broader Deg/ENaC family) function as homotrimers or heterotrimers, suggesting there may be additional Ppk channels involved in mechanosensation in mdIV neurons. Genetic analysis identified Ppk26 and Ppk30 as also expressed in mdIV neurons, and furthermore that they have roles identical to Ppk1 with respect to mechanosensation^8–11^. Here we identify Ppk19 as a new family member dedicated to mechanosensation in mdIV neurons. In addition, we investigate the roles of Ppk family members in acid nociception mediated by mdIV neurons and show that Ppk19, Ppk1, Ppk26, and Ppk30 are required for acid nociception. Thus, these Ppk channels are dedicated to detecting acid and mechanical stimuli in mdIV neurons.

## Results

**Figure 1:**
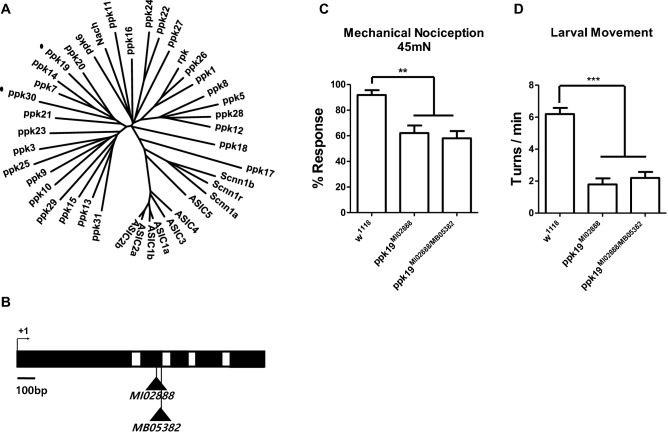
Impaired mechanical nociception and larval movement in *ppk19* mutant larvae. (**A**) Phylogenetic tree of the Ppk family. Ppk19 and Ppk30 are marked with dots. (**B**) MI and MB elements inserted into the second exon of the *ppk19* gene. + 1 denotes the translation start site. The coding sequence and introns are respectively depicted in black and white*.* (**C**) Aversive larval rolling against 45 mN force on larval skin; rolling within two s was counted as response. n > 45 for each genotype. (**D**) Body turns > 40° were counted over a five-minute interval. n > 15 for each genotype. Error bars indicate ± SEM of more than three independent experiments. One-way ANOVA with *Dunnett’s* post hoc test used to test for significant differences. ** and *** indicates *p* < 0.01, *p* < 0.001, respectively.

Phylogenetic analysis revealed that Ppk19 belongs to the subfamily that includes Ppk30 (Fig. 1A), which was previously shown to be required for mechanical nociception and larval movement^11^. This prompted us to examine whether Ppk19 might form ion channels with functions similar to those of Ppk30. Toward this end, we employed P-element insertion lines in which P-elements were inserted in the 2nd exon of *ppk19*, far from the first amino acid, thus probably disrupting protein structure and producing severely hypomorphic mutants (Fig. 1B). *ppk19*^*MI02888*^ homozygotes were viable and presented normal appearance and development. Upon being touched gently with a soft brush, the larvae changed direction, indicating that gentle touch sensation was not affected (Fig S1A); similarly, heat contact on the body caused larvae to aversely roll, indicating normal thermal nociception (Fig S1C). Meanwhile, harsh mechanical force on the skin provoked less aversive rolling (Fig. 1C), indicating impairment of mechanical nociception. Wandering wild-type larva exhibit stereotyped larval movement mediated by mdIV neurons: they move straight with frequent turns^5^. Such larval movements were also impaired (Fig. 1D). Transheterozygote *ppk19*^*MI02888/MB05382*^ larvae also exhibited impaired mechanical nociception and larval movement, but no impairment of thermal nociception and gentle touch (Figs. 1C,D, S1A,C). Taken together, these results support that the Ppk19 ion channel is dedicated to mechanosensation mediated by mdIV nociceptors.

**Figure 2:**
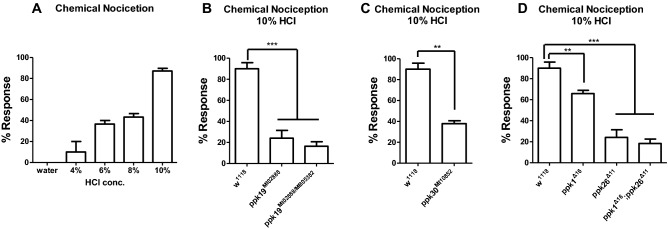
Impaired chemical nociception in *ppk19* mutant larvae. (**A**) Aversive rolling response of wild-type (*w*^*1118*^) larvae in acid solution; rolling within 10 s was counted as response. n = 30 for each concentration. Error bars indicate ± SEM of more than three independent experiments. (**B-D**) Larval rolling in 10% HCl solution; rolling within 10 s was counted as response. n > 45 for each genotype. Error bars indicate ± SEM of more than three independent experiments. One-way ANOVA with *Dunnett’s* post hoc test used to test for significant differences. **, *** indicates *p* < 0.01, *p* < 0.001, respectively.

Acid nociception is also mediated by mdIV neurons^3^; therefore, we tested whether *ppk19* is involved in acid nociception. In accord with a prior report, control (*w*^*1118*^) larvae exposed to acid aversively rolled in a dose-dependent manner (Fig. 2A). This acid nociception was impaired in *ppk19* mutant larvae compared to control (*w*^*1118*^) larvae (Fig. 2B). Transheterozygote *ppk19*^*MI02888/MB05382*^ larvae also exhibited impaired acid nociception (Fig. 2B). In addition, mutants for *ppk1*, *ppk26*, and *ppk30*, genes all previously shown to be required for mechanical nociception and larval movement, also exhibited impaired acid nociception (Fig. 2C,D).

**Figure 3:**
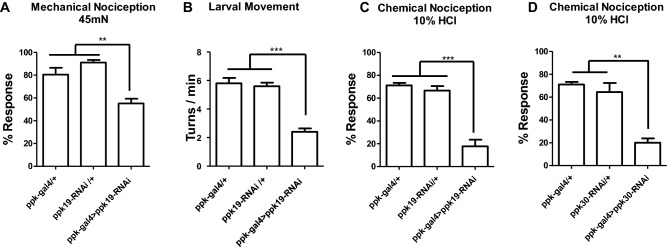
Impaired mechanical and chemical nociception and larval movement with *ppk19* RNAi expression in mdIV neurons. (**A**) Larval rolling against 45mN force on larval skin; rolling within two s was counted as response. n > 45 for each genotype. (**B**) Body turns > 40° were counted over a five-minute interval. n > 15 for each genotype. (**C-D**) Larval rolling in 10% HCl solution; rolling within 10 s was counted as response. n > 45 for each genotype. Error bars indicate ± SEM of more than three independent experiments. One-way ANOVA with *Dunnett’s* post hoc test was used to test for significant differences. ** and *** indicates *p* < 0.01, *p* < 0.001, respectively.

Having established that *ppk19* mutant larvae are impaired in the acid and mechanical sensation mediated by mdIV neurons, we next examined whether *ppk19* might act in mdIV neurons to mediate those sensations. To reduce *ppk19* function in mdIV neurons, cell-specific expression of *ppk19* RNAi (*UAS-ppk19* RNAi) was produced by means of mdIV neuron-specific *gal4* (*ppk-gal4*)^5^. In flies expressing *ppk19* RNAi in mdIV neurons (*ppk-gal4* > *ppk19* RNAi), both acid and mechanical nociception and also larval movement were impaired; meanwhile, neither thermal nociception nor gentle touch were disrupted (Figs. 3, S1B,D). These impairments were partially rescued by expressing *ppk19* in mdIV neurons (Fig S2).

*ppk30* was previously shown to be expressed in mdIV neurons, wherein it mediates mechanical^11^ and acid nociception (Figs. 2C and 3D). We examined the possibility that *ppk19* genetically interacts with *ppk30*. Compared to heterozygotes for either *ppk19* (*ppk19*^*MI02888/*+^) or *ppk30* (*ppk30*^*MI10852/*+^), the transheterozgote (*ppk19*^*MI02888*^*/ppk30*^*MI10852*^) exhibited reduction in mechanical and acid nociception (Fig S3A,C). In contrast, the transheterozygote *ppk19*/*ppk1* or *ppk19*/*ppk26* did not exhibit reduction in mechanical and acid nociception (Fig S3B,D). These suggest that *ppk19* genetically interacts with *ppk30*, but not with *ppk1* or *ppk26*, and hence Ppk19 might form a hetero-complex with Ppk30. To examine whether Ppk19 physically interacts with Ppk30, we employed the fragment complementary assay (FCA), in which N-terminal and C-terminal GFP fragments exhibit fluorescence when they are in close proximity^12^. Transfection with either *ppk19-GFP-N* (N-terminal fragment of GFP) or *ppk30-GFP-C* (C-terminal fragment of GFP) did not produce GFP fluorescence as observed on confocal imaging and measured by fluorescence assay (Fig S4). In contrast, when *ppk19-GFP-N* and *ppk30-GFP-C* were co-transfected into cells, GFP fluorescence was increased (Fig S4).

**Figure 4:**
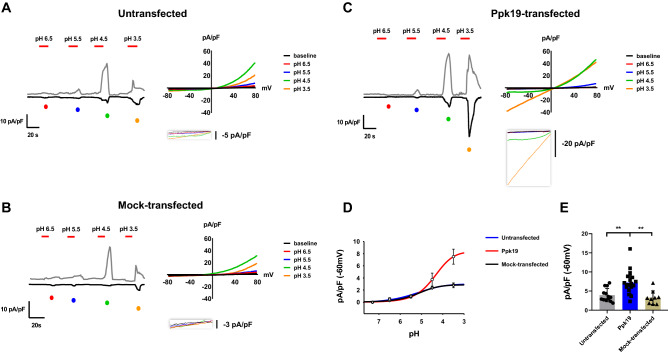
pH dependency of Ppk19-mediated currents. (**A-C**) Representative whole-cell recording traces at ± 60 mV (left) and current–voltage relationships (right) upon extracellular pH stimulation in untransfected (**A**), parental vector (mock)- (**B**) and *ppk19-*transfected CHO-K1 cells (**C**). Currents indicated as dots of different colors below the traces were used to extract current–voltage relationships. Inset: expanded view. (**D**) pH response curves were calculated using the Hill equation from acid responses at − 60 mV for untransfected, *ppk19*-, and parental vector (mock)-transfected CHO-K1 cells. (**E**) Inward currents at − 60 mV during pH 3.5 exposure in untransfected, *ppk19*-, and parental vector (mock)-transfected CHO-K1 cells. **, *p* < 0.01 by unpaired two-tailed *t*-test.

The above genetic analysis indicating that Ppk19 might act as an acid- and mechanosensor prompted us to empirically test this functionality. To examine whether Ppk19 forms ion channels that respond to acid and mechanical force, we carried out whole-cell patch clamp assays in cultured cells. Upon acid stimulus, *ppk19*-transfected cells readily produced inward currents significantly greater than those occurring in either untransfected cells or parental vector (mock)-transfected cells (Fig. 4). Moreover, the magnitudes of inward currents produced from *ppk19*-transfected cells increased in a pH-dependent manner, and *ppk19*-transfected cells exhibited a efficacy (pA/pF) three times that of mock-transfected cells (8.3 *vs.* 3.2) (Fig. 4D,E). In response to acid exposure, Ppk19 channels were permeable to Na^+^ (1.7-fold increase in *ppk19*-transfected cells compared to mock-transfected cells) but impermeable to K^+^, Ca^2+^, and Cl^-^ (Fig S5A,C). Acidic Ppk19 currents were not inhibited by amiloride and benzamil, inhibitors of Deg/ENaC, and were not affected by extracellular Ca^2+^ concentration (Fig S6A,B,E).

**Figure 5:**
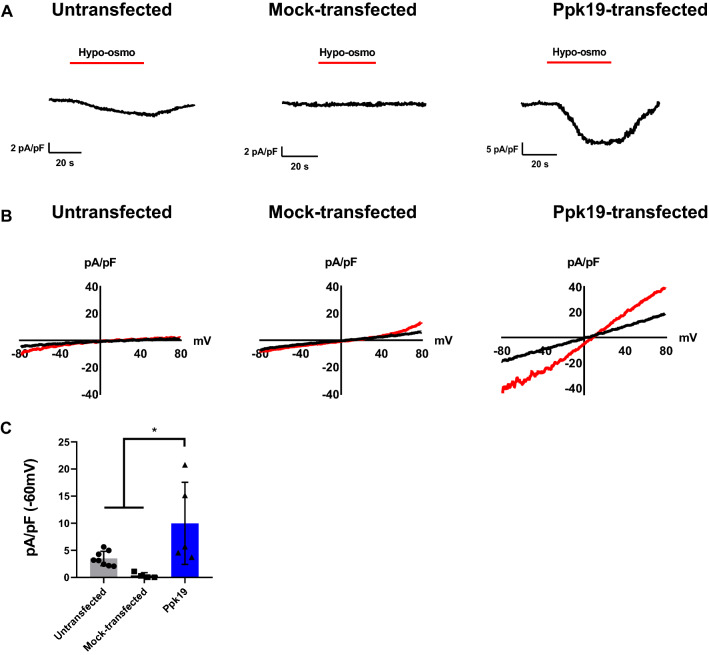
Ppk19 currents in response to osmotic stretching. (**A**) Representative whole-cell currents in patch clamp recordings at − 60 mV during hypotonicity exposure (indicated by “Hypo-osmo”) in untransfected, parental vector (mock)-, and *ppk19*-transfected S2 cells. (**B**) Current–voltage relationships from whole-cell currents in response to hypotonicity-induced mechanical stress in untransfected, parental vector (mock)-, and *ppk19*-transfected S2 cells. (**C**) Currents at − 60 mV during hypotonicity exposure in untransfected, parental vector (mock)-, and *ppk19*-transfected S2 cells. *, *p* < 0.05 by unpaired two-tailed *t*-test.

To examine whether Ppk19 opens in response to mechanical force, *ppk19*-transfected cells were challenged with hypo-osmotic solutions, which induce cell swelling and stretching. Upon osmotic stretching, *ppk19*-transfected cells produced inward currents significantly greater than those produced by either untransfected or parental vector (mock)-transfected cells (Fig. 5). *ppk19*-transfected cells exhibited a efficacy (pA/pF) five times that of mock-transfected cells (9.8 *vs.* 1.7) (Fig. 5C), and the hypo-osmotic Ppk19 currents were reduced by amiloride (Fig S6C,G). In response to cell swelling, Ppk19 channels were more permeable to Na^+^ (1.8-fold increase in *ppk19*-transfected cells compared to mock-transfected cells) than K^+^ (1.4-fold increase), but impermeable to Ca^2+^ and Cl^-^ (Fig S5B,C). Challenge with hyper-osmotic solutions did not induce current in *ppk19*-transfected cells (Fig S6D). Taken together, these findings suggest that in cultured cells, Ppk19 can function as an ion channel that opens to produce currents in response to acid and mechanical force.

## Discussion

These studies reveal the physiological roles of *ppk19* in mechanical nociception, mechanosensitive larval movement, and acid nociception, all properties mediated by mdIV neurons. While *ppk19* expression in mdIV neurons could not be demonstrated in this communication, our data suggest that *ppk19* acts in mdIV neurons as an acid- and mechano-detector. Firstly, specific expression of *ppk19* RNAi in mdIV neurons reduced the nociceptive responses against acid and harsh mechanical force and also incurred aberrant larval movement. Secondly, specific expression of *ppk19* in mdIV neurons rescued *ppk19* mutant phenotypes. Thirdly, *ppk19* genetically and physically interacts with *ppk30*, which was previously shown to be exclusively expressed in mdIV neurons. These findings suggest that Ppk19 acts as a complex with Ppk30 in mdIV neurons to detect acid and harsh mechanical forces.

Moreover, this work reveals that *ppk1*, *ppk26*, and *ppk30*, all of which were previously shown to be specifically expressed in mdIV neurons for mechanical nociception, are required for acid nociception. Thus, mdIV neurons appear to employ at least four Ppk channels to detect acid and mechanical cues. As Ppk proteins are a subset within the Deg/ENaC family, whose members function as homotrimers or heterotrimers, Ppk1, Ppk26, Ppk30, and Ppk19 could form either homotrimers or a combination of heterotrimers with each other. A previous report showed that *ppk1* genetically and physically interacts with *ppk26*^8–10^. In this manuscript we show that *ppk19* genetically and physically interacts with *ppk30*, but not with *ppk1* or *ppk26*. Therefore, our findings suggest that mdIV neurons employ at least two complexes of acid and mechano-detectors: one consisting of a Ppk1-Ppk26 heterotrimer and the other a Ppk19-Ppk30 heterotrimer. This is analogous to heat detectors, of which mdIV neurons employ two: Painless, a TRP ion channel, and Subdued, an anoctamin family ion channel^1,13^. To detect mechanical nociception, mdIV neurons use two types of mechano-detectors: one being a subset of the Ppk family, and the other Piezo^14^.

Our whole-cell patch clamp analysis revealed that Ppk19 induces Na^+^ currents in response to acid, indicating that Ppk19 can form homotrimers that are functional and respond to protons. Similarly, Ppk30 was previously shown to mediate acid-induced currents^11^, indicating that Ppk30 can also form proton-gated homotrimers. Ppk1 might form proton-gated ion channels as well since it was previously shown that *ppk1* mutants are defective in acid-induced currents in mdIV neurons^15^. The proton-gated natures of Ppk19, Ppk30, and Ppk1 are consistent with the impaired acid nociception observed in *ppk19*, *ppk30*, and *ppk1* mutant larvae. Taken together, it can be concluded that these channels constitute acid sensors in mdIV neurons and function to detect acid and mediate acid-induced nociception.

Since the physiological roles of Ppk19, Ppk1, Ppk26, and Ppk30 include mechanical nociception and larval movement, these proteins may also function as mechanosensitive ion channels. To date, no report has demonstrated evidence for such functionality. Here, our whole-cell patch clamp analyses showed that Ppk19 induced currents upon cell swelling. These findings suggest that in cultured cells, Ppk19 forms homotrimers that respond to mechanical force, thereby supporting that *ppk19* encodes a mechanosensitive ion channel.

The acid-sensing ion channels (ASICs) in mammals are a subgroup of the Deg/ENaC family, and constitute Na^+^-sensitive ion channels that respond to acid^16^. In sensory neurons, ASICs are dedicated to the detection of changes in extracellular proton concentration, producing acid-induced nociception^16^. Intriguingly, ASICs are also reported to play roles in mechanical-responsive nociception and proprioception^17–19^. In this communication, we show that Ppk19, a *Drosophila* member of the Deg/ENaC family, mediates Na^+^ current in response to acid and also mediates acid nociception in nociceptive sensory neurons. Moreover, we show that Ppk19 produces currents in response to mechanical stimuli and consistently mediates mechanical nociception and mechanosensitive larval movement. Taken together, our findings indicate that Ppk19 plays a role remarkably similar to that of ASICs in mammalian sensory neurons.

It is worth mentioning the properties of Ppk19 currents. In response to acid and cell swelling, Ppk19 is permeable to Na^+^, but less permeable to K^+^, Ca^2+^ and Cl^-^. These properties are similar to the ASIC channels, which are more permeable to Na^+^ than other ions (except for ASIC1a, which is also permeable to Ca^2+^)^20–24^. We show that the currents produced by Ppk19 upon cell swelling are inhibited by amiloride, an inhibitor of Deg/ENaC channels. Amiloride was also previously shown to inhibit Ripped Pocket (Rpk), a member of the Ppk family^6^, suggesting that inhibition by amiloride constitutes a general property of Ppk family members. However, amiloride did not inhibit currents produced by Ppk19 in response to acid solutions as low as pH 3. Similarly, it did not inhibit Ppk30 currents generated in response to pH 3^11^. In mammals, the currents of the ASIC2a/ ASIC2b heteromer are amiloride-sensitive at pH 7 ~ 5 but insensitive at pH below 3.0^25^. Amiloride insensitivity is also observed in ASIC3, in which acid-induced currents are not inhibited by amiloride at pH 7, but inhibited at pH 6^26^. Taken together, these suggest that inhibition by amiloride can be pH-sensitive for certain Ppk and ASIC channels. The nature of pH dependency of amiloride effects on Ppk and ASIC channels require future scrutiny.

## Methods

### *Drosophila* strains

Strains *w*^*1118*^, *ppk19*^*MI02888*^ (#36434), *ppk19*^*MB05382*^ (#25293), *ppk-gal4*, and *ppk19*-RNAi (#58203, #25887) were sourced from the Bloomington *Drosophila* Stock Center, and *ppk30*-RNAi (#105896) from the Vienna *Drosophila* Resource Center. *ppk1*^*Δ16*^ and *ppk26*^*Δ11*^ were gifted by Yu Nung Jan (UCSF). *UAS-ppk19* transgenic flies were generated by injecting *UAS-ppk19* vector into *phiC31* attP-bearing eggs at the Korea *Drosophila* Resource Center (KDRC).

### Molecular biology

For rescue experiments, *ppk19* cDNA was obtained by reverse transcriptase-polymerase chain reaction (RT-PCR) using TOPscript™ RT DryMIX-dT18 (Enzynomics, RT200) from total RNA extracted from *w*^*1118*^ larvae with the forward primer 5′-ggaatccatgttgctgtacaccaa-3′ and reverse primer, 5′-ggggtaccctacttagtatactctttc-3′. The obtained cDNA was cloned into the SST13-UAS vector^27^ using EcoRI and KpnI sites; the resulting *UAS-ppk19* vector was verified by sequencing. For FCA experiments, *ppk19* CDS lacking the stop codon was amplified by PCR using the forward primer 5′-cgcggatccatgttgctgtacaccaag-3′ and reverse primer 5′-cccccaagcttcttagtatactctttcaAattttatgcg-3′. Similarly, *ppk30* CDS (*ppk30* cDNA (IP12342) obtained from the *Drosophila* Genetic Resource Center) without stop codon was amplified by PCR using the forward primer 5′-gcggatccatgagtgccaccgcctgg-3′ and reverse primer 5′-cccccaagcttggagccatggggtatgcg-3′. The amplified DNA was cloned into vectors containing the N- or C-terminal portion of Kusabira Green Protein (CoralHue® Fluo-chase Kit, AM-1100) using BamHI and HindIII sites and the sequence was verified. For electrophysiology experiments, the *ppk19* CDS was cloned into pIRES2-EGFP (Addgene) to produce the pIRES2-EGFP-*ppk19* vector. Briefly, the *ppk19* CDS was amplified by PCR using the forward primer 5′-gactagttatgttgctgtacaccaag-3′ and reverse primer 5′-ccgaattcctacttagtatactctttc-3′. The amplified DNA was then cloned into pIRES2-EGFP using SpeI and EcoRI sites and the sequence was verified.

### Fragment complement assay (FCA)

Vectors were transfected into HEK293T cells. After 48 h, GFP fluorescence was detected by confocal microscopy (Leica TCS SPE) or measured with a fluorescence spectrophotometer (Molecular Devices, SpectraMax GeminiXPS). In confocal images, GFP fluorescence was obtained at 500 nm excitation and at 550 nm emission. For GFP quantification, the vectors were co-transfected with pCMV-ß-gal. GFP fluorescence units (excitation 494 nm, emission 538 nm) were normalized with ß-gal activity units according to the manual. The quantification was repeated twice for triplicate samples.

### Thermal nociception assays

Larval thermal nociception assays were performed as previously described^1^. Briefly, third instar larvae were placed on 3% agarose medium in 55 × 12 mm plastic Petri dishes, and their abdominal segments were touched with a soldering iron having a 0.6 mm-wide chisel tip. The iron’s temperature was measured with an electronic thermometer. A complete roll of 360° along the body axis within ten s of heat exposure (42 °C) was scored as aversive behavior. Each larva was tested only once (n > 45, 15 larvae per test).

### Mechanical nociception assays

Larval mechanical nociception assays were performed as previously described^1^. Briefly, third instar larvae were stimulated using a 45 mN calibrated Sulon monofilament fishing line (6 lb test, diameter 0.23 mm, length 18 mm) that was attached to a 200 ul yellow tip. Noxious mechanical stimuli were delivered by rapidly depressing larvae with the fiber on the dorsal side. A complete roll of 360° along the body axis within two s of mechanical exposure was scored as aversive behavior. Each larva was tested only once (n > 45, 15 larvae per test).

### Gentle touch assay

Touch sensation assays were performed as previously described^28^. Briefly, the mouthparts of third instar larvae were touched gently with a fine brush. Larva response was scored using the Kernan system, defined as follows: 0 = no response, 1 = pausing mouth-hook movement, 2 = withdrawing or turning away from the touch, 3 = reverse peristaltic wave, and 4 = multiple peristaltic waves away from the touch. Each larva was tested only once (n > 45, 15 larvae per test).

### Chemical nociception assay

Larval chemical nociception assays were performed as previously described^3^. Briefly, a concentrated HCl stock solution (37%, EMSURE^Ⓡ^100,317) was diluted to 4–10%. Individual feeding-third instar larvae were placed on a 3% agarose plate and exposed to acid by dropping 2 ul of a diluted HCl solution on the larva’s posterior end. A complete roll of 360° along the body axis within ten s of HCl exposure was scored as aversive behavior. Each larva was tested only once (n > 45, 15 larvae per test).

### Larval movement test

Larval movement assays were performed as previously described^8^. Briefly, wandering third instar larvae were placed on a 3% agarose plate; five minutes later, larval movement was observed. Each larva was recorded with a digital camera for five minutes, and was tested only once (n > 15, 5 larvae per test). Body turns > 40° were counted over a 5 min interval.

### Cell cultures and transfections

Whole-cell patch clamp recording was performed using Chinese hamster ovary-K1 (CHO-K1) cells and *Drosophila melanogaster* S2 cells. Glass coverslips (35-mm diameter) were coated with Poly-L-ornithine for one hour, after which cells were seeded on the coating. CHO-K1 cells were transiently transfected with pIRES2-EGFP plasmids (Addgene) containing *ppk19* cDNA using the Neon Nucleofector System (Thermo Fisher Scientific) according to the manufacturer’s protocol. S2 cells were transiently transfected with pAc5.1A plasmids (Invitrogen) containing *ppk19* cDNA. CHO-K1 cells were incubated in a 5% CO_2_ incubator at 37 °C, and S2 cells were maintained in a room-temperature incubator. At 20–24 h after transfection, the coverslips were put in a recording bath chamber and recordings of the cells with green fluorescence were conducted.

### Whole-cell patch clamp recordings

Whole-cell currents were measured using a MultiClamp 700B Microelectrode Amplifier at a holding potential of –60 mV (Molecular Devices) at room temperature. Recording pipets were pulled from borosilicate glass to a final resistance of 2–4 MΩ and their tips were fire-polished. Only cells with seal resistances over 3–10 GΩ were used for recording. After establishing a whole-cell configuration, currents were recorded by applying 200 ms voltage-ramp pulses from − 80 to + 80 mV every 750 ms. At these given voltages, we observed any change in the currents produced upon stimulation by acid or hypotonic stress as mentioned below.

Currents were digitized with a Digidata 1440A converter (Molecular Devices), filtered at 5 kHz, and analyzed using Clampfit 10.5 (Molecular Devices). The internal pipette solution contained 140 mM CsCl, 5 mM EGTA, 10 mM HEPES (titrated to pH 7.2 with CsOH). The extracellular bath solution contained 140 mM NaCl, 5 mM KCl, 2 mM CaCl_2_, 1 mM MgCl_2_, 10 mM glucose and 10 mM HEPES (titrated to pH 7.3 with NaOH). For extracellular acid stimulations, an acidic bath solution was perfused; this consisted of 140 mM NaCl, 5 mM KCl, 2 mM CaCl_2_, 1 mM MgCl_2_, 10 mM glucose and 10 mM MES (titrated to pH 3.5 with HCl). For extracellular pH 3.5 acidic bath solution with or without Ca^2+^ was used 2 mM EGTA instead of 2 mM CaCl_2._ Hypotonic stress experiments used an extracellular isotonic bath solution containing 91 mM NaCl, 5 mM KCl, 2 mM CaCl_2_, 1 mM MgCl_2_, 10 mM glucose and 10 mM HEPES, and 98 mM mannitol (titrated to pH 7.3 with NaOH) and a stress solution incorporating the same substances but without mannitol, as previously described. Hypertonic stress experiments used an extracellular bath solution containing 142 mM mannitol. Amiloride, benzamil was dissolved in extracellular bath solution and used at 200 µM, 300 µM, respectively.

To avoid the electrical masking that occurs in mammalian cells due to endogenous swelling stress-activated currents, only S2 cells were used for hypotonic stress experiments. For determination of the permeability ratios of other cations versus Cs^+^, reversal potentials were obtained and analyzed using the following Goldman-Hodgkin-Katz equations: E_reversal_ = (RT/zF)ln(P_Na_[Na]o/P_Cs_[Cs]i) or E_reversal_ = (RT/z)ln{√(4P_Ca_[Ca]o/P_Cs_[Cs]i + 1/4)-1/2}. Cation permeability experiments used extracellular bath solutions containing 100 mM CaCl_2_, 10 mM HEPES, and 150 mM NaCl or KCl, 10 mM HEPES for monovalent cation. For determination of the ratio of Cl^-^ versus Cs^+^ permeability (P_Cl_/P_Cs_), reversal potentials of the currents produced under exposure to external 400 mM NaCl and internal 150 mM NaCl were obtained and the following Goldman-Hodgkin-Katz equation applied: E_reversal_ = (RT/zF)ln({[Cs]o + [Cl]iP_Cl_/P_Cs_}/{[Cs]i + [Cl]oP_Cl_/P_Cs_). Potencies and Kd values regarding pH sensitivity were calculated using the Hill equation: current = maximal current efficacy/(1 + (Kd/[H^+^]))^n^.

## Supplementary Information

Supplementary Information.

## Data Availability

The datasets used and/or analysed during the current study available from the corresponding author on reasonable request.
