# Supplementary material for: Drosophila ppk19 encodes a proton-gated and mechanosensitive ion channel
Source: Sci Rep. 2022 Nov 1;12:18346. doi: 10.1038/s41598-022-23236-3 (PMC9626565; doi:10.1038/s41598-022-23236-3)
Supplement: Supplementary file 1 — Supplementary Information. [file 41598_2022_23236_MOESM1_ESM.pdf]

***Drosophila ppk19* encodes a proton-gated and mechanosensitive ion channel**

Supplementary figure legends

Figure S1. Gentle touch and thermal nociception assays. (**A,B**) The gentle touch assay measures response of larvae to gentle touch on the mouthparts, coded as suggested by Kernan [1]. 0: no response, 1: pause (hesitate), 2: recoil (anterior withdraw), 3: single reverse contractile wave, 4: multiple waves of reverse contraction. n>45 for each genotype. (**C,D**) The thermal nociception assay measures larval rolling against a heat probe (44 °C) on the skin; rolling within 10 seconds was counted as response. n =105 for w^{1118}, n>45 for other genotypes. Error bars indicate ±SEM of more than three independent experiments. One-way ANOVA with *Dunnett's* post-test used to test for significant differences.

Figure S2. Rescue of *ppk19* mutant defects with expression of *ppk19* in mdIV neurons. Rescue denotes *ppk-gal4*/+;*UAS-ppk19, ppk19*$^{MB05382/\,MI02888}$. (**A**) Rescue of mechanical nociception. The response percentage was measured by counting the number of larvae showing rolling upon 45 mN of mechanical force on larval skin. n>45 for each genotype. (**B**) Rescue of larval movement. Body turns >40° were counted over a five-minute interval. n>15 for each genotype. (**C**) Rescue of chemical nociception, measured as larval nociceptive response against noxious chemical (10% HCl) on larval skin. n>90 for w^{1118}, n>45 for other genotypes. Error bars indicate ±SEM of more than three independent experiments. One-way ANOVA with *Dunnett's* post-test used to test for significant differences. *, *** indicates $p<0.05$, and $p<0.001$, respectively.

Figure S3. Genetic interaction between *ppk19* and *ppk30*. (**A,B**) Mechanical nociception assay. Percent response was counted as larval roll within 2 sec of 45 mN force on larval skin. n>45 for each genotype. (**C,D**) Chemical nociception assay. Percent response was counted as larval roll within 10 sec of 10% HCl drop on larvae. n>45 for each genotype. Error bars indicate ±SEM of more than three independent experiments. One-way ANOVA with Dunnett's post-test was used to test for significant differences. ** and *** indicates $p<0.01$, $p<0.001$, respectively. *ppk19*^{MI02888}/*ppk30*^{MI10852} denotes transheterozygotes for *ppk19*^{MI02888} and *ppk30*^{MI10852}.

Figure S4. Physical interaction between Ppk19 and Ppk30. (**A**) Confocal images of GFP fluorescence. (**B**) GFP fluorescence as measured by fluorescence spectrophotometer. GFP fluorescence level was normalized to β-gal level. Ppk19-GFP_N, Ppk30-GFP_C denote ppk19-mKG_N and ppk30-mKG_C in which mKG_N and mKG_C indicate the N-terminal and C-terminal fragments of Kusabira Green Protein.

Figure S5. Ion permeabilities accompanying induced Ppk19 currents. (**A**) Permeabilities of Na^+, K^+, Ca^{2+}, and Cl^- in *ppk19*-transfected CHO-K1 cells upon pH 3.5. (**B**) Permeabilities of Na^+, K^+, Ca^{2+}, and Cl^- in *ppk19*-transfected S2 cells upon hypo-osmotic pressure (200 mOsm/kg). (**C**) Summary of Ppk19 ion permeabilities. Permeabilities were calculated from the reversal potentials of basal currents with CsCl in the internal solution and the indicated chloride salt in the external solution. *, $p<0.05$ by unpaired two-tailed *t*-test.

Figure S6. Characteristics of Ppk19-mediated induced currents. (**A-E**) Representative current-voltage relationships of *ppk19*-transfected CHO-K1 cells upon exposure to acid (**A,B,E**), hypo-osmolarity (**C**), hyper-osmolarity (**D**). Amiloride (200 µM) and benzamil (300 µM) are featured (**A,B**). (**F,G**) Efficacy (pA/pF) at -60 mV for *ppk19*-transfected CHO-K1 cells upon exposure to acid (**F**) and for *ppk19*-transfected S2 cells in response to hypo-osmolarity (**G**), with drug treatment and Ca^{2+} concentration as indicated**.** **, $p<0.01$ by unpaired two-tailed *t*-test.

References

1 Kernan, M., Cowan, D. & Zuker, C. Genetic dissection of mechanosensory transduction: mechanoreception-defective mutations of Drosophila. *Neuron* **12**, 1195-1206, doi:10.1016/0896-6273(94)90437-5 (1994).

Fig S1

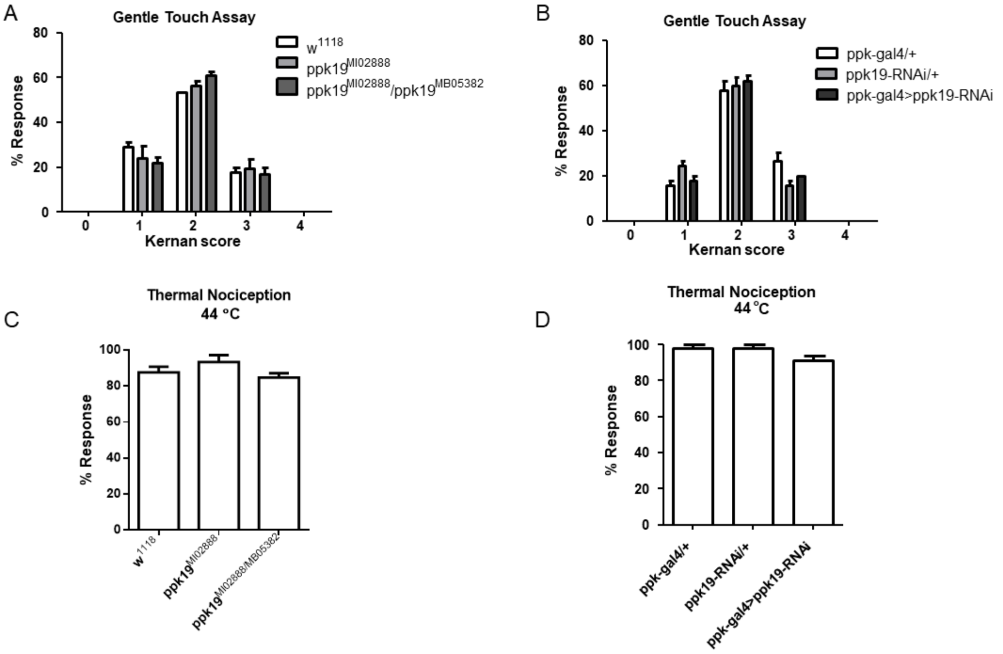
A
Gentle Touch Assay
% Response
Kernan score
w1118
ppk19MI02888
ppk19MI02888/ppk19MB05382
B
Gentle Touch Assay
% Response
Kernan score
ppk-gal4/+
ppk19-RNAi/+
ppk-gal4>ppk19-RNAi
C
Thermal Nociception
44 °C
% Response
w1118
ppk19MI02888
ppk19MI02888/MB05382
D
Thermal Nociception
44 °C
% Response
ppk-gal4/+
ppk19-RNAi/+
ppk-gal4>ppk19-RNAi

Fig S2

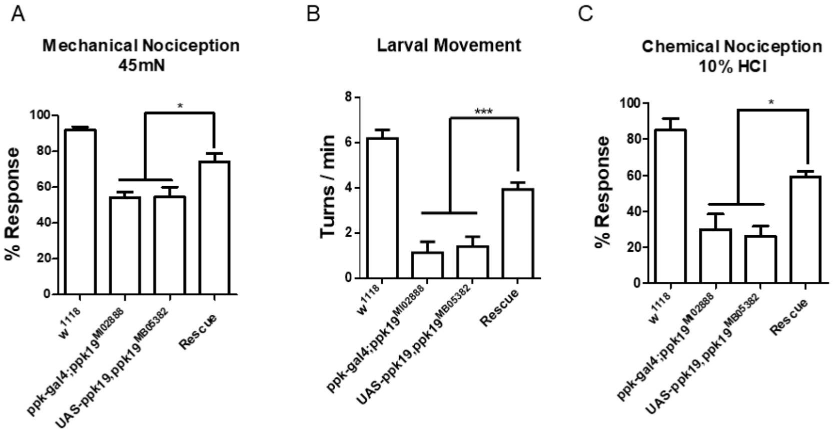
A
Mechanical Nociception
45mN
% Response
100
80
60
40
20
0
*
w 1118
ppk-gal4;ppk19 MI02888
UAS-ppk19,ppk19 MB05382
Rescue
B
Larval Movement
Turns / min
8
6
4
2
0
***
C
Chemical Nociception
10% HCl

Fig S3

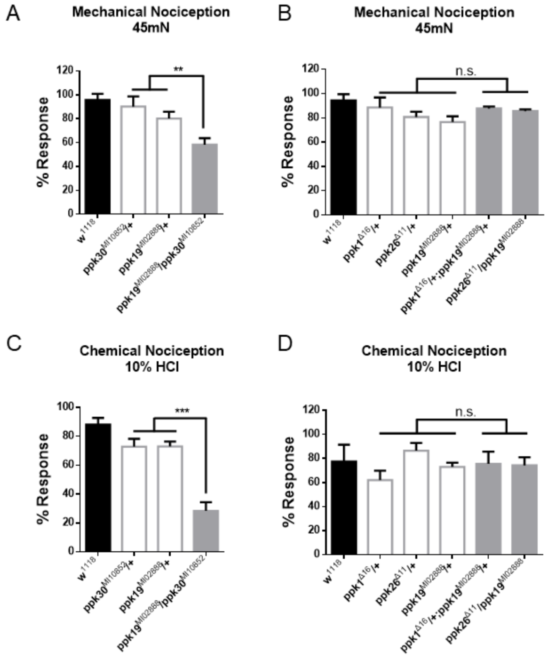
A
Mechanical Nociception
45mN
% Response
**
w1118
ppk30MI10852/+
ppk19MI02888/+
ppk19MI02888/ppk30MI10852
B
Mechanical Nociception
45mN
% Response
n.s.
w1118
ppk1Δ16/+
ppk26Δ11/+
ppk19MI02888/+
ppk1Δ16/+;ppk19MI02888/+
ppk26Δ11/ppk19MI02888
C
Chemical Nociception
10% HCl
% Response
***
w1118
ppk30MI10852/+
ppk19MI02888/+
ppk19MI02888/ppk30MI10852
D
Chemical Nociception
10% HCl
% Response
n.s.
w1118
ppk1Δ16/+
ppk26Δ11/+
ppk19MI02888/+
ppk1Δ16/+;ppk19MI02888/+
ppk26Δ11/ppk19MI02888

Fig S4

A

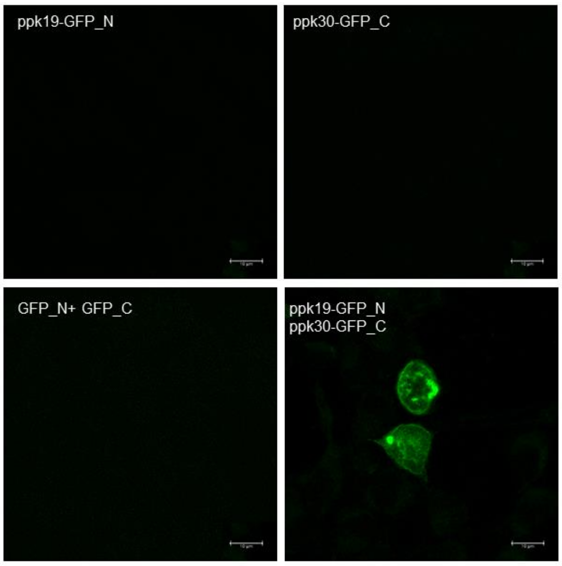
ppk19-GFP_N
ppk30-GFP_C
GFP_N+ GFP_C
ppk19-GFP_N
ppk30-GFP_C

B

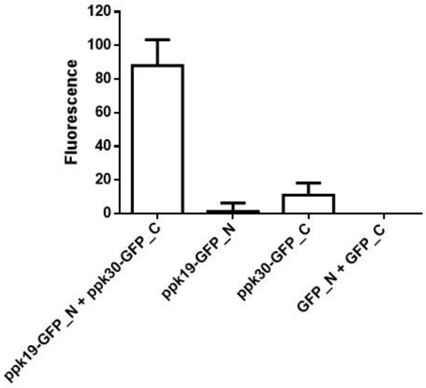
Fluorescence
120
100
80
60
40
20
0
ppk19-GFP_N + ppk30-GFP_C
ppk19-GFP_N
ppk30-GFP_C
GFP_N + GFP_C

Fig S5

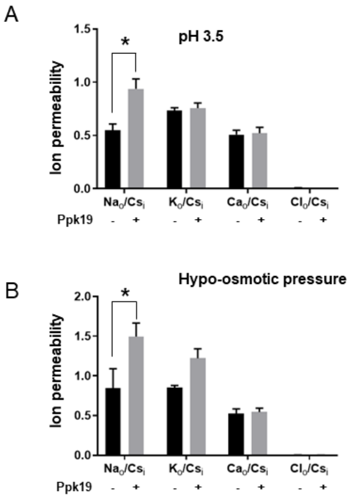

C

Ion permeability (P_x/P_{cs})	Ppk19-transfected		Mock-transfected	
	pH 3.5	Hypo-osmotic pressure	pH 3.5	Hypo-osmotic pressure
Na^+	0.94±0.07 (n=5)	1.50±0.19 (n=5)	0.55±0.06 (n=5)	0.85±0.33 (n=4)
K^+	0.76±0.05 (n=6)	1.22±0.13 (n=6)	0.73±0.03 (n=8)	0.85±0.03 (n=6)
Ca^{2+}	0.52±0.06 (n=5)	0.55±0.05 (n=5)	0.51±0.04 (8)	0.52±0.07 (n=7)
Cl^-	0.002±0.0003 (n=9)	0.002±0.0006 (n=5)	0.008±0.001 (n=8)	0.002±0.003 (n=6)

Fig S6

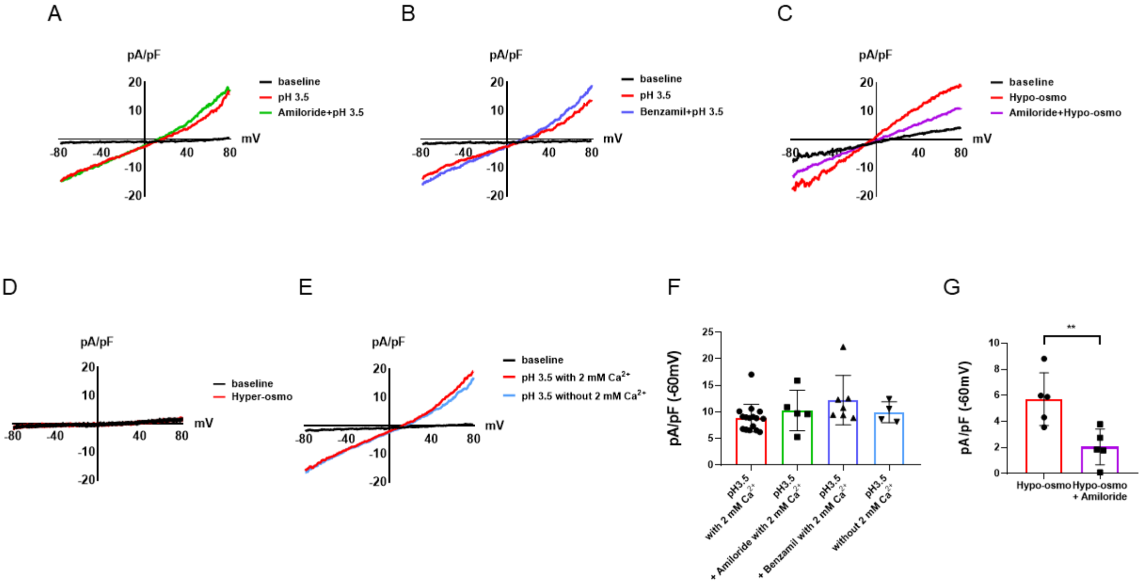
A
pA/pF
mV
baseline
pH 3.5
Amiloride+pH 3.5
B
Benzamil+pH 3.5
C
Hypo-osmo
Amiloride+Hypo-osmo
D
Hyper-osmo
E
pH 3.5 with 2 mM Ca2+
pH 3.5 without 2 mM Ca2+
F
pA/pF (-60mV)
pH3.5 with 2 mM Ca2+
pH3.5 + Amiloride with 2 mM Ca2+
pH3.5 + Benzamil with 2 mM Ca2+
pH3.5 without 2 mM Ca2+
G
pA/pF (-60mV)
**
Hypo-osmo
Hypo-osmo + Amiloride
